# Caspofungin Use in Daily Clinical Practice for Treatment of Invasive Aspergillosis: Results of a Prospective Observational Registry

**DOI:** 10.1186/1471-2334-10-182

**Published:** 2010-06-22

**Authors:** Johan Maertens, Gerlinde Egerer, Wan Shik Shin, Dietmar Reichert, Michael Stek, Sheenu Chandwani, Malathi Shivaprakash, Claudio Viscoli

**Affiliations:** 1 UZ Gasthuisberg, Leuven, Belgium; 2 Universitätsklinikum Heidelberg, Heidelberg, Germany; 3 Department of Internal Medicine, The Catholic University of Korea College of Medicine, Seoul, Korea; 4 Klinikum Oldenburg gGmbH, Oldenburg, Germany; 5 ID Consultant Services, Ambler, PA, USA; 6 UMDNJ- Robert Wood Johnson Medical School, Department of Family Medicine- Research Division, Somerset, NJ, USA; 7 Merck & Co., Inc. Whitehouse Station, NJ, USA; 8 San Martino University Hospital, Genova, Italy

## Abstract

**Background:**

A prospective observational registry assessed real world experience with caspofungin monotherapy or combination therapy for the initial or salvage treatment of proven or probable invasive aspergillosis (IA).

**Methods:**

Data were collected from April 2006 to September 2007 for patients treated with caspofungin for a single episode of IA. Clinical effectiveness was categorized as favorable (complete or partial) or unfavorable (stable disease or failure) at the end of caspofungin therapy (EOCT).

**Results:**

Consecutive patients (n = 103) with proven or probable IA (per EORTC/MSG criteria) were identified from 11 countries. Malignancy (76.7%), neutropenia (64.1%), allogeneic hematopoietic stem cell transplantation (HSCT, 22.3%), solid organ transplantation (8.7%), autologous HSCT (4.9%), and HIV/AIDS (2.9%) were the most common underlying conditions. Most patients (84.5%) had pulmonary IA. *Aspergillus fumigatus *was the most frequently isolated species. The majority of patients received caspofungin monotherapy (82.5%) primarily as salvage therapy (82.4%). The main reason for switching to salvage therapy was clinical failure of the first-line therapy (69%). A favorable response at EOCT was seen in 56.4% (57/101) of patients overall, including 56.5% (48/85) and 56.3% (9/16) of patients receiving caspofungin monotherapy and combination therapy, respectively. Favorable response rates in clinically relevant subgroups were: malignancy, 51.9% (41/79); allogeneic HSCT, 56.5% (13/23); and neutropenia at time of hospitalization, 53.0% (35/66). There was a 72.3% (73/101) survival at 7 days after EOCT. Serious adverse events related to caspofungin were reported in 4 cases (3.9%); 3 patients (2.9%) discontinued treatment due to an adverse event related to caspofungin.

**Conclusions:**

Caspofungin was both effective and well tolerated among high-risk patient groups such as those with neutropenia and active malignancies.

## Background

Invasive aspergillosis (IA) accounts for approximately 30% of all invasive fungal infections [1]. In spite of recent improvements in our ability to diagnose the disease at an earlier stage and the availability of new antifungal drugs, IA still carries a high mortality rate [2,3]. In addition, in carefully selected study patients with proven, probable or possible IA, overall response rates are approximately 50% for both voriconazole (52.8%), a current drug of choice, and for liposomal amphotericin B (46-50%), the currently recommended alternative for first-line therapy [1,4,5]. Response rates are considerably lower among allogeneic hematopoietic stem cell transplant recipients (HSCT) than in patients with active hematologic malignancies [4,5].

Caspofungin is the first echinocandin approved for use in the treatment of IA in patients who are refractory to or intolerant of other agents. In this setting, caspofungin monotherapy has an overall success rate of 45% to 60% in the context of clinical trials and outside clinical trials in an open case setting [6-9]. However, there is a need for continuing studies of caspofungin efficacy and tolerability because the efficacy of these agents may change over time and is likely to vary in different real-world practices compared with clinical trials. This article describes a prospective observational registry developed to assess daily clinical practice with caspofungin when used as monotherapy or in combination therapy for initial or salvage treatment of proven or probable IA.

## Methods

A prospective observational registry was developed to collect data from consecutive patients treated with caspofungin monotherapy or caspofungin combination therapy for proven or probable IA. The protocol was approved by the institutional review boards at each participating center. Data collection was conducted in accordance with the ethical standards outlined in the declaration Helsinki and guidelines for good clinical practices [10].

### Patient Population

Only patients older than 16 years of age who were receiving caspofungin for treatment of proven or probable IA and who were not currently participating in another Merck sponsored clinical trial for invasive fungal infections were eligible. Investigators identified eligible patients based on their diagnosis of aspergillus infection and categorized their infection based on their clinical judgment and local practice standards. The sponsors of the study provided guidance to investigators for proven or probable *Aspergillus *infection diagnosis according to the 2002 European Organisation for Research and Treatment of Cancer/Mycoses Study Group (EORTC/MSG) criteria [11]. Patients with radiologic signs highly suggestive of aspergillosis were eligible for inclusion. There were no protocol-defined exclusion criteria.

### Registry Design

Given the observational nature of this study, no investigational or approved medication was provided to participating centers. All patients were treated according to the standard of care at the participating center. Data were collected between April 2006 and September 2007 across 23 sites in 11 countries including Australia, Belgium, Brazil, Germany, Greece, Jordan, Korea, Russia, Singapore, Slovenia, and Taiwan.

Response was assessed by the investigators at the end of caspofungin therapy (EOCT) using standard definitions [11]. A complete or partial response at EOCT was considered a favorable response; stable disease, failure (progression of disease), or death from any cause was considered an unfavorable response. Complete response required resolution of all attributable clinical signs and symptoms and complete resolution of radiographic or bronchoscopic abnormalities. Partial response required clinically meaningful improvement in all attributable clinical signs and symptoms, a significant improvement of radiographic (at least 50%) or bronchoscopic abnormalities, and included persistence of radiographic sequelae regardless of the overall level of clinical or radiographic improvement. Stable disease included no improvement of attributable clinical signs or symptoms and no improvement of radiographic or bronchoscopic abnormalities. Failure included deterioration in attributable clinical or radiographic abnormalities that necessitated alternative antifungal therapy or resulted in death.

The safety evaluations required all investigators to report serious and non-serious clinical or laboratory adverse event that was related to the administration of caspofungin. Serious adverse events were those resulting in death or incapacity, were life-threatening, required initial or prolonged hospitalization, and included birth defects, cancer, and those deemed to be serious by medical judgment. An independent expert review panel consisting of two experts (Dr. J. Maertens and Prof. C. Viscoli) reviewed the analysis of the data for accuracy and completeness.

### Statistical Analysis

Patient demographics and baseline patient characteristics were summarized. Patients were categorized according to whether they received caspofungin as monotherapy or whether azoles or polyenes were administered in combination with caspofungin. Summary statistics stratified by monotherapy and combination therapy were also provided for first-line versus second-line use of caspofungin. Caspofungin effectiveness and safety were assessed by the number and percentage of patients with a favorable response at the EOCT and adverse event reports; 95% confidence intervals were calculated for effectiveness data. Effectiveness was also evaluated for various patient sub-groups including proven vs probable disease; caspofungin monotherapy vs combination therapy; caspofungin as first-line therapy vs salvage therapy; neutropenic status at caspofungin initiation (<500 cells/μL vs ≥ 500 cells/μL); and risk factors at baseline. Additional summary statistics were provided for length of hospital stay (LOS), survival at 7 days after EOCT, and microbiology.

## Results

A total of 103 patients with proven (n = 31; 30.1%) or probable (n = 72; 69.9%) IA was enrolled in the registry. The mean age of the population was 50.4 years and the male-to-female ratio was 2:1 (Table 1). Two patients (2%) were excluded from the effectiveness analysis because they were still on study therapy at the time the database was closed and therefore were not assessed. Pulmonary IA was the most frequent manifestation of infection, accounting for 84.5% of cases (n = 87), followed by blood infection (fungemia) (7.8%, n = 8). Other sites of infection included bone/joint, sinus, liver/spleen, external ear and trachea (one case each). Three patients had disseminated infection (2.9%). The main predisposing conditions included malignancy (76.7%) and allogeneic HSCT (22.3%), followed by solid organ transplantation (8.7%), autologous HSCT (4.9%), and HIV/AIDS (2.9%). Many patients presented with multiple predisposing factors with a mean number of 5.3 (SD= 1.78) risk factors present per patient; 57% of patients were neutropenic (ANC<500 cells/μL) at the start of caspofungin treatment.

**Table 1:** Patient demographics and baseline characteristics

Variable	Monotherapy (N = 85)	Combination therapy (N = 18)	Overall (N = 103)
Gender, n (%)			
Male	55 (64.7%)	11 (61.1%)	66 (64.1%)
Female	30 (35.3%)	7 (38.9%)	37 (35.9%)

Mean age (years) (mean ± SD^a^)	49.8 ± 15.94	53.4 ± 16.94	50.4 ± 16.09

Race, n (%)			
White	49 (57.6%)	15 (83.3%)	64 (62.1%)
Asian	36 (42.4%)	3 (16.7%)	39 (37.9%)

Country, n (%)			
Germany	36 (42.4%)	6 (33.3%)	42 (40.8%)
Korea	28 (32.9%)	3 (16.7%)	31 (30.1%)
Russia	8 (9.4%)	0	8 (7.8%)
Taiwan	6 (7.1%)	0	6 (5.8%)
Greece	0	5 (27.8%)	5 (4.8%)
Other	7 (8.2%)	4 (22.2%)	11 (10.7%)

Mean APACHE II score (mean ± SD)	18.3 ± 8.26 (n = 11)	18.3 ± 2.52 (n = 3)	18.3 ± 7.31 (n = 14)
SOFA score (mean ± SD)	8.8 ± 4.66 (n = 5)	0	8.8 ± 4.66 (n = 5)

Site of Infection, n (%)			
Blood	8 (9.4%)	0	8 (7.8%)
Lung	72 (84.7%)	15 (83.3%)	87 (84.5%)
Bone/joint	1 (1.2%)	0	1 (1.0%)
Sinus	0	1 (5.6%)	1 (1.0%)
Liver/spleen	0	1 (5.6%)	1 (1.0%)
Other	2 (2.4%)	0	2 (1.9%)
Multiple	2 (2.4%)	1 (5.6%)	3 (2.9%)

Neutropenic at time of initiation of caspofungin treatment, n (%)			
<500 cells/μL	53 (62.4%)	6 (33.3%)	59 (57.3%)
>500 cells/μL	32 (37.6%)	12 (66.7%)	44 (42.7%)

Number of risk factors per patient (mean ± SD)	5.4 ± 1.76	4.8 ± 1.86	5.3 ± 1.78

Risk factors and underlying medical conditions^b^, n (%)			
Active malignancy	70 (82.4%)	9 (50%)	79 (76.7%)
Immunosuppressive medication	64 (75.3%)	12 (66.7%)	76 (73.8%)
Neutropenia at time of hospitalization	59 (69.4%)	7 (38.9%)	66 (64.1%)
Allogeneic HSCT	18 (21.2%)	5 (27.8%)	23 (22.3%)
Prior fungal colonization	16 (18.8%)	2 (11.1%)	18 (17.5%)
Diabetes mellitus	11 (12.9%)	4 (22.2%)	15 (14.6%)
Organ transplantation	5 (5.9%)	4 (22.2%)	9 (8.7%)
Autologous HSCT	5 (5.9%)	0	5 (4.9%)
AIDS/HIV infection	3 (3.5%)	0	3 (2.9%)

^a^SD = standard deviation

^b^Frequencies of different risk factors and underlying medical conditions are not mutually exclusive.

The mean duration of caspofungin therapy was 18.9 ± 21.4 days (range 1-40.3 days). The mean daily dose of caspofungin was 49.3 ± 6.97 mg. In 42 patients, no attempt was made to have a culture-based diagnosis. Of the 61 patients in which culture examination was performed, 34 tested negative, although these patients were identified as having proven/probable IA by the investigator, according to the other criteria (such as positive histopathological evidence), as defined in the EORTC/MSG guidelines. In culture-positive cases (n = 27, 26.2%), *Aspergillus fumigatus *was the most frequently isolated species (n = 10), followed by *A. flavus *(n = 3); 9 cases yielded a positive culture for *Aspergillus *but did not have the species identified. Supportive microbiological data were missing in 5 cases; diagnosis in these cases was based on the investigator's judgment. The majority of patients received caspofungin as salvage therapy (83/103 = 80.6%) (Table 2). Seventy of these patients received monotherapy and 13 patients received caspofungin-containing combination treatment (usually with voriconazole). The main reasons for switching to salvage therapy included clinical failure of the first-line therapy (69%), microbiological documentation of persistence of the infection (8.4%), or toxicity associated with the first-line therapy (10.8%). Antifungals administered prior to switching to caspofungin were amphotericin B deoxycholate (30.1%), fluconazole (29.1%), itraconazole (18.4%), voriconazole (12.6%) or a lipid-based formulation of amphotericin B (11.7%). Twenty patients (19.4%) received caspofungin as first-line monotherapy (n = 15) or combination therapy (n = 5).

**Table 2:** Patients receiving caspofungin treatment

Indication for IV caspofungin therapy	Monotherapy (N= 85) n (%)	Combination therapy (N = 18) n (%)	Overall (N = 103) n (%)
Caspofungin first-line therapy	15 (17.6)	5 (27.8)	20 (19.4)

Caspofungin salvage therapy	70 (82.4)	13 (72.2)	83 (80.6)
Clinical refractory to first-line antifungal	48 (68.6)	10 (76.9)	58 (69.0)
Microbiological refractory to first-line antifungal	6 (8.6)	1 (7.7)	7 (8.4)
Toxicity			
• Nephrotoxicity to first-line antifungal	2 (2.9)	1 (7.7)	3 (3.6)
• Other toxicity	6 (8.6)	0	6 (7.2)
Other*	7 (10.0)	1 (7.7)	8 (9.6)
Not reported	1(1.4)	0	1 (1.2)

*Other reasons include: Severe disease requiring combination therapy, prophylaxis, elevated *Aspergillus *antigen, empirical therapy, elevated creatinine

### Response at EOCT

An overall favorable response rate of 56.4% (57/101) was observed in this study population. Patients receiving caspofungin monotherapy and combination therapy had response rates of 56.3% (9/16) and 56.5% (48/85), respectively (Table 3). Those receiving first-line therapy had a slightly higher response rate (60.0%) than those receiving salvage therapy (55.6%). Caspofungin first-line monotherapy was associated with a 60.0% (9/15) favorable response. Favorable response rates in clinically relevant subgroups included a 51.9% response rate in patients with active malignancy, 56.5% in those with allogeneic HSCT and 53.0% in patients with neutropenia (Table 3).

**Table 3:** Favorable response (complete plus partial) by patient subgroup (N = 101)

Variable	Favorable response % (n/N) [95%CI]
Overall	56.4 (57/101) [46.7; 66.1]

Probable aspergillosis	56.3 (40/71) [44.0; 68.1]
Proven aspergillosis	56.7 (17/30) [37.4; 74.5]

Combination therapy	56.3 (9/16) [29.9; 80.2]
First line	60.0 (3/5) [14.6; 94.7]
Second Line	54.6 (6/11) [23.4; 83.3]
Monotherapy	56.5 (48/85) [45.3; 67.2]
First line	60.0 (9/15) [32.3; 83.7]
Second Line	55.7 (39/70) [43.3; 67.6]

First-line therapy	60.0 (12/20) [36.1; 80.9]
Salvage therapy	55.6 (45/81) [44.1; 66.6]

Culture examination performed	62.3 (38/61) [50.1; 74.5]
Positive	55.6 (15/27) [36.9; 76.6]
Negative	67.6 (23/34) [49.5; 82.6]

Neutropenic status at start of caspofungin therapy	
ANC < 500 cells/μL	52.5 (31/59) [39.1; 65.7]
ANC > = 500 cells/μL	61.9 (26/42) [45.6; 76.4]

Risk factors	
Active malignancy	51.9 (41/79) [40.4; 63.3]
AIDS/HIV infection	66.7 (2/3) [9.4; 99.2]
Bone marrow/stem cell transplantation	53.6 (15/28) [33.9; 72.5]
• Autologous HSCT	40.0 (2/5) [5.3; 85.3]
• Allogeneic HSCT	56.5 (13/23) [34.5; 76.8]
Diabetes mellitus	57.1 (8/14) [28.9; 82.3]
Immunosuppressive medication	60.8 (45/74) [48.8; 72.0]
Neutropenia at the time of hospitalization	53.0 (35/66) [40.3; 65.4]
Prior fungal colonization	61.1 (11/18) [35.7; 82.7]
Organ transplantation	75.0 (6/8) [34.9; 96.8]

Response evaluated in N = 101 patients.

### Outcomes

The overall LOS was 59.9 days. Increased LOS due to adverse events was observed in 1 patient (1.0%), and increased LOS due to treatment failure with caspofungin was observed in 7 patients (6.8%). Survival rates of 74.1% (63/85) were observed at 7 days after the end of caspofungin monotherapy and 62.5% (10/16) at 7 days after the end of combination therapy.

### Safety

No patient discontinued caspofungin therapy due to drug interactions between caspofungin and other antifungal drug. According to the investigator, 3/103 patients (2.9%) experienced a serious drug-related clinical adverse event (bronchopneumonia, respiratory failure, skin reaction), including two patients (1.9%) who discontinued caspofungin therapy (Table 4). One patient (1.0%) developed a serious laboratory adverse event (hyperbilirubinemia) that resulted in discontinuation of caspofungin therapy. Twenty-three patients (27.1%) in the monotherapy and 5 patients (27.8%) in the combination therapy group died. No reports were received from investigators attributing any death to caspofungin therapy.

**Table 4:** Adverse events

Type of Adverse Events	Clinical Adverse Events* N = 103 n (%)	Laboratory Adverse Events** N = 103 n (%)
Any adverse events	4 (3.9)	8 (7.8)
Serious adverse events	3 (2.9)	1 (0.9)
Serious adverse events leading to discontinuation	2 (1.9)	1 (0.9)

* Clinical adverse events include: bronchopneumonia; skin reaction; respiratory failure; and abdominal pain

**Laboratory adverse events include: increase in aspartate aminotransferase, alanine aminotransferase, blood alkaline phosphatase, blood bilirubin, gamma glutamyltransferase; leukopenia; hyperbilirubinemia; and hypokalemia.

## Discussion

Caspofungin regimens provided high favorable response and survival rates in this observational registry documenting real world findings. An overall favorable response rate of 56.3% was observed in patients with proven or probable aspergillosis treated with caspofungin monotherapy or combination therapy. A total of 20/101 patients received caspofungin as first-line therapy (as part of a combination regimen in 5 patients), which was associated with a 60.0% favorable response. Favorable responses >50% were seen in high risk patient subgroups, including those with a malignancy (51.9%), with allogeneic HSCT (56.5%) or neutropenia at the time of hospitalization (53.0%). There was a 73% survival at 7 days after EOCT. A low rate of drug-related serious adverse events and drug-related adverse events leading to discontinuation was observed, with no differences noted between the safety profiles of caspofungin monotherapy and combination therapy.

The high rates of favorable response observed in this registry were impressive considering the severity of illness in the patient population. The majority of patients in this study (81.6%) required salvage therapy due to prior treatment failure or toxicity. The patients included in this registry were very ill at baseline, as evidenced by the high proportion of patients with neutropenia (57.3%) and active malignancies (76.7%). Additionally, some patients received caspofungin after failing amphotericin B therapy due to lack of efficacy or intolerance. Therefore, it should be considered that long-term persistence of relevant amphotericin B concentration in organ tissue may have led to effectiveness not solely attributable to caspofungin. It is also worth noting that survival was lower for patients who received combination therapy as this group was likely composed of a sicker population with a greater disease burden in comparison to those selected for monotherapy.

The favorable effectiveness and survival results presented in this registry were consistent with the cumulative published literature on caspofungin. The registry data confirm the previously reported efficacy of caspofungin monotherapy [6] and combination therapy [7] in clinical trials. The favorable response rates and survival rates in our study reflected trends observed with liposomal amphotericin B and voriconazole when used as first-line therapy, although there was a difference in the time to follow up between this and the published studies [4]. The median duration of therapy was also shorter in this study than in clinical studies of caspofungin therapy (median, 25 days, range, 1-196 days; median, 28 days; range, 1-162 days) [6,7]. In a double-blind trial of patients (n = 201) with invasive mold infections, 97% of whom had IA, 50% of patients achieved a favorable response with liposomal amphotericin B and 72% survived to at least 12 weeks [5]. A randomized, unblinded study of patients (n = 144) with IA found that 52.8% of patients who received voriconazole had a favorable response, compared with only 31.6% who received amphotericin B; 12-week survival rates of 70.8% and 57.9% were achieved in the voriconazole and amphotericin B groups, respectively [4]. A prospective database study of 85 patients with allogeneic HSCT, 24 with graft versus host disease, found an overall response rate of 31% with amphotericin B lipid complex as first- or second-line therapy [12].

The present registry had several inherent limitations. As an observational registry documenting real world use of caspofungin in proven and probable IA, no comparator arm was available and no superiority or non-inferiority could be determined. In addition, the low numbers of patients in some of the categories may have skewed the results. While deaths occurring during the observation period were determined to be unrelated to caspofungin, the reasons for death were not collected with the study data. Diagnosis of proven and probable IA was based on investigators' clinical judgment and local practice standards using the EORTC/MSG criteria as a guide. All sites were not mandated to provide comprehensive diagnostic information and treatment practices for IA, which may have varied between countries and regions. This could have resulted in misclassification of disease. Cases with changes highly suggestive of IA on radiology were eligible for study inclusion as probable IA, whereas EORTC/MSG criteria would categorize these cases as possible IA. Therefore, possible cases could have been included as probable cases resulting in a bias away from null and hence, an overestimation of response rates. As an observational study, reports of fungemia were accepted as recorded by investigators and all patients with proven or probable IA were included. Many patients with *A. fungemia *have a low performance score and exhibit co-morbidities which excludes them from clinical studies, but not from registries; therefore, the number of fungemia cases in our study may have been greater than expected. Finally, there was a limited follow-up period after caspofungin therapy ended.

## Conclusions

In this observational registry of daily clinical practice, antifungal regimens in which caspofungin was used as monotherapy or combination therapy, and as either first-line or second-line therapy, provided effective treatment in severely ill patients with proven or probable IA. Caspofungin was both effective and well tolerated among high-risk patient groups such as those with neutropenia and active malignancies.

## List of Abbreviations

EOCT: End of caspofungin therapy; EORTC/MSG: European Organisation for Research and Treatment of Cancer/Mycoses Study Group; HSCT: Hematopoietic stem cell transplantation; IA: Invasive aspergillosis; SD: Standard deviation

## Competing interests

J. Maertens has received research grants from Merck/MSD and Pfizer; is a consultant to Astellas, Bio-Rad, Merck/MSD, Nektar, Pfizer, Schering-Plough, F2G, and Zeneus/Cephalon; and has served at the speakers' bureau of Astellas, Bio-Rad, Merck/MSD, Pfizer, Schering-Plough, and Zeneus/Cephalon.

G. Egerer has received research funding from MSD SHARP & DOHME GmbH and Schering-Plough; has been a member of the speaker's bureau of MSD, SHARP & DOHME GmbH and Schering-Plough; and has received travel grants from MSD SHARP & DOHME GmbH and Schering-Plough.

M Stek was an employee of Merck & Co. Inc. (manufacturer of caspofungin) when the study was designed and since retirement from the company has served as a consultant to Merck & Co., Inc.

S. Chandwani is currently an employee of UMDNJ. At the time of the study she was participating in a joint fellowship between Merck & Co., Inc. and Rutgers University.

M. Shivaprakash is an employee of Merck & Co., Inc, which manufactures caspofungin.

C. Viscoli has served on speakers' bureaus for Merck, Pfizer, Schering-Plough, and Gilead; has served on advisory boards for Merck, Pfizer, Schering-Plough, Gilead, and Astellas; and has received research grants from Gilead, Abbott, Boeheringer-Ingelheim, and Pfizer. P.G.P. has received research grants from Merck, Pfizer, SPRI, and Astellas; has served on speakers' bureaus for Merck, Pfizer, and Astellas; and has been an ad hoc advisor for Merck, SPRI, Pfizer, Astellas, Novartis, and Eisi

## Authors' contributions

SC and MS had full access to all of the data in the study and take responsibility for the integrity of the data and the accuracy of the data analysis. JM, MS, MS, and CV conceived the study and participated in its design. JM, GE, WSS, DR, and MS were responsible for acquisition of data. JM, MS, SC, MS, and CV were responsible for interpretation of data. MS and SC participated in drafting the manuscript. All authors participated in critical revision of the manuscript for important intellectual content. MS was responsible for study supervision. All authors read and approved the final manuscript. CAN-DO(IA) study team was responsible for contributing patients in the study.

## Pre-publication history

The pre-publication history for this paper can be accessed here:

http://www.biomedcentral.com/1471-2334/10/182/prepub
